# New Concepts in the Investigation and Treatment of Prostatic Hyperplasia

**Published:** 1992-12

**Authors:** J. McLoughlin

**Affiliations:** Senior Urological Registrar


### New Concepts in the Investigation and Treatment of Prostatic Hyperplasia

**J. McLoughlin, M.S., F.R.C.S.**
Senior Urological Registrar

*Address for Correspondence:*
Bristol Royal Infirmary
Marlborough Street
Bristol BS2 8HW

As the development of benign enlargement of the prostate is age related, the number of patients presenting with "prostatism" or urinary retention can be expected to increase as the population ages. This has huge financial implications for the National Health Service. Clinicians dealing with such patients need not only to be able to objectively assess men whose symptoms suggest bladder outflow obstruction but also be in a position to tailor their treatment in order to respect the wishes of those who want to avoid side effects such as retrograde ejaculation, impotence or the risk of blood transmitted infection that may accompany traditional treatments.

With such a common problem it may appear surprising that a number of fundamental difficulties remain. Firstly there are no universally accepted means of assessing possible candidates for prostatectomy. With regards to symptoms, all may be unreliable, although hesitancy is possibly the most reliable symptom. Most urologists now employ flow rate measurements, often coupled with estimation of residual urinary volume, however the value of formal urodynamic evaluation for patients with "uncomplicated prostatism" or whether they improve the post-operative outcome still remains unanswered.

In assessing the outcome of therapy, a number of earlier studies employed little more than flow rate measurements to determine the presence of obstruction. *Carter et al.* (1992 ) recently observed statistically significant improvements in flow rate studies can be seen on the second and third attempts, as patients learnt how to "produce" a flow rate. Apart from the diagnostic implications this finding is of importance in relation to the assessment of patients following a period of treatment for prostatism where improvements in flow rates may be the only objective measurement employed to determine relief of obstruction.

A common feature of alternatives to prostatectomy is that they produce a degree of symptomatic relief, often marked, but with only modest improvement in objective parameters of obstruction, the actual improvement in flow rates for example being only in the order of a few ml./sec.. This would imply a degree of residual obstruction remaining. The question as to whether it is enough to reduce symptoms without achieving urodynamic improvement remains controversial, as it is by the resolution of his symptoms that a patient will gauge the success of his treatment.

Another consideration is the non acquisition of prostate tissue for histology following non surgical therapy allows the possibility that small foci of prostate cancer may go undiagnosed, these patients potentially being denied a curative operation by non-diagnosis. The placebo effect on the outcome of treatment of patients with "prostatism" may account for 30% of men improving in some studies (*Castro et al.*, 1971). This point will later be expanded in relation to the specific modalities employed.

While the most frequent treatment is transurethral resection of the prostate (TURP) not all men want an operation. *Kaplan et al.* (1992) recently divided men according to the severity of their symptoms, reporting that patients generally preferred a more conservative approach when offered an alternative, those with mild or moderate symptoms choosing medical therapy while those with more troublesome symptoms or retention preferring minimally invasive procedures. TURP or "watchful waiting" were not the main choice in any patient group.

As the mechanism of obstruction is both a static effect due to the bulk effect of the prostatic mass and, in addition, a dynamic effect as a result of the muscular component under adrenergic stimulation, the following account will use this model as a means of rationalising treatment options.

### TREATMENTS AVAILABLE

### THERMAL DISRUPTION OF PROSTATIC TISSUE

#### Hyperthermia and Thermotherapy

Applying heat to the prostate, primarily directed at the peri-urethral transitional zone, produces a spectrum of structural alterations accompanied by symptomatic and urodynamic improvement. The distinction between hyperthermia and thermotherapy lies in the temperature generated within the heated tissue. With local hyperthermia, tissue is heated to 42 - 45 degrees centigrade, the optimum temperature being around 43 degrees. The results obtained are directly related to the time for which treatment is applied. Hyperthermia regimes may thus involve as many as 5 or 10 outpatient sessions (as with the Biodan Prostathermer or Primus systems respectively), each lasting 1 hour. Benign hyperplastic tissue is largely unaffected at these temperatures with no histological alteration observed.

Thermotherapy (also termed Trans-urethral Microwave Thermotherapy - TUMT), such as that employed by the Prostatron system, achieves temperatures of between 45 - 60 degrees centigrade, the urethra being protected by a urethral cooling system incorporated into the catheter. At these higher temperatures, hyperplastic prostatic tissue undergoes clearly identifiable oedema and subsequent necrosis, this being substituted ultimately with connective tissue resulting in a reduction of the static obstructive element. The degree to which prostatic tissue is disrupted is reflected by the rise in PSA that accompanies therapy - up to 890% in some series (*Perrin et al.*, 1992) and by MRI images showing a cavity comparable to that following TURP produced by a single session of thermotherapy (*Tazaki*, 1991). In addition to tissue destruction, receptors or their connections may be destroyed, thereby affecting the dynamic obstructive component.

Hyperthermia series report between 50 - 76% symptomatic improvement and 46 - 63% improvement in flow rates. It appears to be especially beneficial for patients with irritative symptoms (*Bdesha et al.*, 1992). However, the actual level of improvement in flow rates are often modest, for example *Ludac et al.* (1992) reporting only 0.9 and 1.2 ml. / sec. increase at 12 weeks and 6 months over baseline values. The use of thermotherapy in patients presenting with acute retention have been encouraging with 50% achieving spontaneous voiding (*Beavan et al.*, 1992).

The potential placebo effect of hyperthermia has been investigated, *Zerbib et al.* (1992) employing a treatment versus sham group. While both groups experienced symptomatic improvement (68% in the treatment group compared to 38% in the sham group), improvement in objective parameters were only observed in the treatment group. These findings have subsequently been confirmed by *Bdesha et al* (1992) in a similar prospective, controlled treatment versus sham trial. On the basis of these studies it would appear that hyperthermia produced effects greater than those of placebo. Patients tolerate hyperthermia well, with urgency and bladder spasms being the most frequent complaint (*Beart et al.*, 1992) although in one series 18% of men required analgesics per-operatively (*Ludac et al.*, 1992). Adverse effects have typically included mild haematuria (41%), local pain (37%), transient dysuria (19%) and urinary infection (19%) (*Beart et al.*, 1992). Hyperthermia is reported to have minimal effects on fertility, with no change in ejaculatory volume, seminal characteristics or retrograde ejaculation. A common finding through hyperthermia and thermotherapy reports is the high post-operative retention rate, occurring in as many as 26 - 30% of men (*Ludac et al.*, 1992).

Investigators have identified those patients most likely to benefit from thermotherapy and it appears that the actual anatomy of the prostate may influence outcome. *Baert et al.* (1992) report that of patients with urinary retention treated by hyperthermia, 18/25 men with bi-lobed prostates voided following treatment compared to only 1 of 7 with median lobe enlargement. *Soldo et al.* (1992) identified patients with large prostates (possibly precluding effective temperature distribution) or decompensated detrusors as poor candidates. Applying selection based on such information, the future success rate may be expected to improve.

Ultimately the clinical value of a treatment such as thermotherapy will be determined by the durability of clinical response. At present little is known after the first 18 months and with the failure of treatments such as balloon dilatation in the longer term this information will be of importance. A major disadvantage of thermotherapy remains the expense of the initial outlay of the generator system.

### Laser therapy

A recent addition is the laser. Laser therapy produces coagulation necrosis resulting in cystic degeneration and fibrosis, ultimately producing a reduction in prostate volume and hence the static component of the obstruction. Initially laser ablation was employed using a transurethral ultrasound guided laser system (TULIP), *McCullough et al.* (1992) reporting 63% reduction in symptoms and 52% improvements in flow rates at 6 months. More recently, systems such as the Neodymium: YAG laser have employed a *lateralase fibre*, the tip of which has a deflecting mechanism to direct the laser energy at 90 degrees which has the major advantage to a Urologist that it can be used in conjunction with a standard 23 F cystoscope mounted with a catheterising port. Laser ablation has been associated with marked symptomatic improvement (symptoms scores falling from 15 to 4) but only modest increases in flow rate (from 5 to 9 ml./ sec.) (*Costello et al.*, 1992). Included in this series were two men with chronic retention and overflow incontinence and one patient with acute retention who voided post-operatively.

A feature of laser treatment is that it is fast, *Costello et al.* (1992) reporting lasing times of only 4.2 minutes. In addition it is accompanied by virtually no blood loss allowing patients to be allowed home the same day with a non-irrigating catheter for 48-72 hours in order to allow resolution of oedema. Present technology has lead exponents of laser therapy to restrict treatment to prostates of <30 gm..

A consideration is that the fibre delivery systems have only a finite life span and their replacement may ultimately limit the cost effectiveness of this treatment.

### MECHANICAL REDUCTION IN PROSTATIC RESISTANCE

### Prostatic Stents

Stents are best thought of as either permanent or temporary. Permanent stents have been constructed of stainless steel superalloys (*McLoughlin et al.*, 1990; *Chapple et al.*, 1990) or titanium (*Gillatt et al.*, 1992) woven into a self expanding mesh. The majority of patients receiving these stents have been those for whom TURP was not indicated because they were either medically unfit or had terminal disease with a limited prognosis (*McLoughlin et al.*, 1990). A feature of these stents are their "pores" which allow epithelialisation, by 3 months in 60% of cases (*Gillatt et al.*, 1992). On this basis the risk of encrustation and stone formation are reduced. Encrustation has not been a major problem with titanium ASI stent in a clinical setting (*Gillatt et al.*, 1992) but has been reported in relation to the Urolume stent (formerly the Wallstent) (*McLoughlin et al.*, 1992). In their recent series, *Milroy* and *Chapple* (1992) observed encrustation on 14 / 30 stents that had been in situ for over 9 months. Encrustation tends to be associated with chronic infection, poor emptying or wires left protruding into the bladder lumen (*McLoughlin et al.*, 1992; *Milroy* and *Chapple*, 1992). Expandable stents have been shown to allow spontaneous micturition in up to 95% of patients following placement (*McLoughlin et al.*, 1990) and follow-up studies have found these to be an acceptable alternative in the long term. *Gillatt et al.* (1992) treated 94 men, followed up over a mean period of 21 months. Of 77 alive, 68 were reported to be voiding well with peak flows of 13ml./sec. and low residual volumes.

While both types of stents are self expanding, modifications have been made including the use of an endoscopically placed balloon to "bed in" the device in order to minimise the risk of displacement and possibly (by producing a snugger fit) also reduce the potential for stone formation.

A disadvantage of permanent stents has been their expense compared to their temporary counterparts.

Of temporary prostatic catheters, the intra-prostatic coil is possibly the best known. This was originally made of steel but later modified by gold plating (Prostakath). In common with other stents, prostatic coils have been shown to relieve retention in 80% of men (*Harrison* and *De Souza*, 1990). Longer term complications include stent displacement and recurrent retention in addition to those experienced with permanent stents (*Rosenkilde et al.*, 1992) indeed *Van Poppel et al.* (1991) found that coils were liable to dislocate at any time following placement. A second temporary stent, the Intra-urethral Catheter (Puroflex -Urosoft), has recently appeared. This resembles a double mallecot 16F polyurethane catheter with a crown at the proximal end (*Nissenkorn* and *Slutzker*, 1991). Attached to the distal end is a non absorbable suture that allows its extraction simply by grasping the suture and pulling. In their series, *Nissenkorn* and *Richter* (1990) report results comparable with other stents, 79% of men with retention voiding spontaneously following catheter placement. It's major advantage is that it is inexpensive. Temporary stents have been used both in those considered unfit for TURP and more recently as a temporary means of avoiding a catheter for men who developed urinary retention after major surgery or a myocardial infarct. They probably need to be changed every 6 months in order to avoid stone formation if used over long periods.

All indwelling prostheses have the potential for urinary infection in much the same way as do urethral catheters. In the absence of any symptoms the major risk of these infections being an increased risk of encrustation and subsequent stone formation (*Holmes et al.*, 1992).

### Balloon Dilatation

Interest in balloon dilatation of the prostate arose in the wake of balloon angioplasty and was spurred on by the prospect of preservation of antegrade ejaculation, low peroperative blood losses and the potential of an outpatient based procedure.

The exact mechanism of dilatation is unclear but may involve either the production of a commissurotomy between prostatic lobes, thereby decompressing the encircling prostatic urethra, or one of a neuropraxia leading to denervation of the prostatic urethra. Progressively larger balloons have been employed in an attempt to produce a more reliable disruption of the commissures (*McLoughlin et al.*, 1991; *Reddy et al.*, 1992) in an effort to produce better results. The failure to do so has lead some investigators to challenge the relative importance of the commissurotomy to a successful outcome (*McLoughlin et al*., 1991; *Jones et al*., 1992).

The success rate of balloon dilatation has varied widely, ranging from improvement in urodynamic parameters in 48% of men at 3 months, this figure falling to 11% and 7% at 6 and 9 months respectively (*McLoughlin et al*., 1991) to 94% at 3 months, 95% of these remaining successful at 1 year (*Klein*, 1992). In common with earlier work undertaken by the author, others have failed to demonstrate sustained benefit (Jones et al., 1992; Roy et al., 1992) with as few as 25% of men remaining symptomatically improved at 2 years (*Jones et al*., 1992).

A criticism of earlier balloon designs was their failure to dilate the whole of the gland. On this basis recent interest has focussed on "measure-to-fit" placed under endoscopic control in order to ensure that the full length length of gland is dilated. To date, little objective evidence has appeared to support claims that such modifications are of any benefit.

As with other alternatives to prostatectomy, the symptomatic improvement experienced is greater and of longer duration than the objective improvement in urodynamic criteria. *McLoughlin et al*. (1991) observed 70% symptomatic improvement at 3 months, this figure being maintained at 55% and 52% at 6 and 9 months respectively despite deterioration in urodynamic parameters. The disparity between symptomatic and urodynamic improvements suggest that a placebo effect may underlie the success of balloon dilatation. To date, only *Lepor et al*. (1992) have undertaken a randomised double-blind trial, comparing cystoscopic controlled balloon dilatation with that of a placebo cystoscopy. Both patient groups experienced statistical improvement in symptom scores (38% for cystoscopy compared to 53% with balloon dilatation) but in the absence of any real increase in flow rate (mean increase 2.4 ml. / sec. in the cystoscopy group and 2.3 ml. / sec. in the balloon group). Such is the interest in balloon dilatation that remains among American urologists, this article recently provoked intense debate in the urological literature.

### MEDICAL THERAPY

### Pharmacological reduction in muscular tone

For some time the use of $\alpha$ blockers have been employed to reduce sympathetic tone, and thereby the dynamic component of obstruction in patients with prostatism. There have been several generations of these drugs including phenoxybenzamine, Prazosin and more recently Indoramin. A feature of each generation has been increased $\alpha 1$ receptor specificity thereby allowing a reduction in side effects such as postural hypotension. Alpha blockade can be expected to produced symptomatic improvement in approximately 70% of men and appear to work best on primary stromal hyperplastic glands. In keeping with other alternatives to prostatectomy, it produces a disproportionate improvement in symptoms compared to that in urodynamic parameters. As these drugs are generally given in divided doses attention has recently turned to longer acting selective $\alpha 1$-blockers such as Terazosin (*Lepor et al*., 1992), Afluzosin (*Teillac et al*., 1992) and Doxazosin (*Carter et al*. 1992; *Chapple et al*., 1992) that allow once daily dosages. Their effects have been demonstrated to be dose dependent (*Teillac et al*., 1992).

Whilst a large volume of work has appeared relating to the short benefits, only recently has information relating to long term administration now appeared. In one such study, patients were evaluated over a 24 month period of Terazosin administration (*Lepor et al*., 1992). Of the 45 men entered, 21 men (47%) exhibited a consistent clinical response over this period. Obstructive and irritative symptom scores decreased by 63% and 35% respectively after 2 months treatment, these improvements being maintained over the 2 year period. Peak and maximum flow rates increased by 42% and 48% respectively after the first 2 months and, while peak flow values decreased slightly after 6 months of therapy, the mean flow rate remained increased over baseline values throughout the study period. An interesting point to arise from this article was that clinical benefit appears relatively quickly and failure to achieve a favourable response within 2 months should lead to discontinuation of treatment.

A number of other placebo controlled studies relating to alpha blockade, for example Terazosin (*Brawer et al*., 1992) or other agents such as Doxazosin (*Carter et al*., 1992) have now appeared in the literature confirming an improvement in flow rates and symptoms over that of placebo.

### Pharmacological reduction on prostatic size

5 $\alpha$ reductase inhibitors serve to reduce the glandular component and thereby the static component of outflow obstruction. These agents are probably best suited to glands with a predominantly glandular hyperplastic component. They act by competitively inhibiting the conversion of testosterone to its active component dihydrotestosterone and have been shown to suppress intra-prostatic levels of dihydrotestosterone by up to 92% within one week of commencing therapy (*Wilson et al*., 1989). As they do not affect testosterone levels, they do not impair libido as did earlier attempts at hormonal manipulation. Using these agents, investigators observed an overall reduction in prostate volume of between 14% and 17% (*Kirby et al*., 1992 and *Tempany et al*., 1992 respectively), *Tempany et al*. (1992) reporting this to be predominantly of periurethral zonal tissue. Similar findings were reported by *Stoner et al* (1992) who evaluated men after 12 weeks therapy and then repeated the assessment after discontinuation of treatment for 12 weeks. All had evidence of a reduction in prostate volume on therapy followed by a return to pre-treatment values after stopping treatment.

*Kirby et al*. (1992) reported a study of 69 men entered into a double blind placebo-controlled study. Patients received either placebo or alternatively 5mg or 10 mg doses of the active drug. All 3 groups of patients experienced symptomatic improvement, however, urodynamic improvement (increased flow rates and reduced voiding pressures) were observed only in those men receiving the active drug. In much the same way as for $\alpha$ blockade, the increase in flow rates have been modest (1.5 ml. / sec. on 10 mg and 3.3 ml. / sec. in 5 mg group). As these drugs act by effecting a reduction in prostatic size, it may take as long as 12 months before full therapeutic effect is achieved (*Kirby et al*., 1992) .

### THE LARGER PROSTATE

A feature of newer approaches have always been their attention to the smaller prostate, larger glands or those with median lobe enlargement remaining largely the domain of TURP. However, patients with large prostate may yet be proven to be best served by open prostatectomy. *Lewis et al* (1992) compared patients undergoing TURP with those treated by an open operation. It was of interest that while the overall complication rate in the first week was much higher in the open compared to the TURP group (38.5% and 17.5% respectively), the late complications occurring in the TURP group were far higher (17.5%) compared to the open group (1.5%), the overall figure being equal. *Crowley et al*. (1992) addressed the question of cardiovascular events following prostatectomy. Over a mean follow up period of approximately 5 years, 95% of the TURP group were alive compared to 77% of the open group. However of interest was that in the TURP group, 50% of the deaths that occurred were due to cardiac causes compared to only 17% in the open group. A number of reports have recently highlighted significant cardiovascular alterations occurring at the time of TURP which may in part account for some of these findings.

### THE FUTURE

As the number of alternatives to prostatectomy increase so does the likelihood of tailoring treatment to an individual patient's requirements.

By generating a range of temperatures with thermotherapy, symptomatic relief without tissue destruction at lower temperatures for the younger patient with symptoms and minimal obstruction may become an option. Alternatively, higher temperatures could be applied to the older patient with retention to produce a TUR-like cavity. Improvements in laser fibre - delivery systems may allow more scope for the bigger glands.

A range of biocompatible plastics are at present under evaluation and may in time reduce the cost of prostatic stents, if only by market pressures. Alternatively the future development of biodegradable prostatic stents may allow outlet decompression for long enough to effect to recovery of the decompensated detrusor.

The future selection of patients for either $\alpha$ blockage and 5 $\alpha$ reductase inhibition may not be a random choice, but rather one made on the basis of information obtained by either transrectal ultrasound scan or prostatic biopsies at the time of initial presentation. Alternatively medical management may involve combining both $\alpha$ blockage and 5 $\alpha$ reductase inhibitors in order to produce rapid symptomatic relief coupled with longer term effects aimed at reversing the underlying development of the hyperplastic gland.

### REFERENCES

Baert, L., Ameye, F., Pike, M. C., Willemen, P., Astrahan, M. A. and Petrovich, Z. (1992). Transurethral hyperthermia for benign prostatic hyperplasia patients with retention. *J. Urol.*: **147**: 1558 -1561.

Beavan, A. J., Ogden, C., Reddy, P., Patel, A., Ramsey, J.W. A., and St. Carter, S. (1992). Treatment of urinary retention with transurethral microwave therapy (T.U.M.T.). 114A Proceedings of the British Association of Urological Surgeons, Bournemouth.

Bdesha, A. S., Bunce, C., Snell, M. E., Vukusic, J. and Witherow, R. O'N. (1992). A controlled trial of transurethral hyperthermia in prostatism. 168A. Proceedings of the British Association of Urological Surgeons, Bournemouth.

Brawer, M., Epstein, H., Adams, G., Henry, D. and Clifton, C. (1992). Efficacy and safety of Terazosin in patients with symptoms of benign prostatic hyperplasia: A new double-blind study. *J. Urol.*: **147**: 611A.

Carter, P. G., Lewis, P. and Abrams, P. (1992) The flow clinic and value of multiple flow rates. 41A Proceedings of the British Association of Urological Surgeons, Bournemouth.

Carter, P., Chapple, C. R., Christmas, T.J., Noble, J. G., Miller, P., Kirby, R. S., Abrams, P. and Milroy, E. J. G. (1992). A 3 month double-blind placebo study of Doxazosin for BPH. 162A Proceedings of the British Association of Urological Surgeons, Bournemouth.

Castro, J. E., Griffiths, H. J. L. and Edwards, D. E. (1971). A double blind controlled clinical trial of Spironolactone for benign prostatic hypertrophy. *Br. J. Surg.*: **58**: 485 - 489.

Chapple, C. R., Milroy, E. J. G. and Rickards, D. (1990). Permanently implanted urethral stent for prostatic obstruction in the unfit patient. Prelimary report. *Brit. J. Urol.*: **66**: 58 - 65.

Chapple, C. R. and Milroy, E. J. G. (1992). Permanent Urolume prostate stent: 3 year experience-Indications and problems. *J.Urol.*: **147**: 379A.

Chapple, C. R., Carter, P., Christmas, T. J., Noble, J. G., Miller, P., Kirby, R. S., Abrams, P. and Milroy, E. J. G. (1992). A three month double-blind placebo controlled study of Doxazosin as treatment for benign prostatic bladder outlet obstruction. *J. Urol.*: **147**: 613A.

Crowley, A. R., Horowitz, M. and Macchia, R. J. (1992). Transurethral resection of prostate versus open prostatectomy : Mortality comparison in patients without prior medical illness. *J. Urol.*: **147**: 615A.

Costello, A. J., Bowsher, W. G., Bolton, D. M., Braslis, K. G. and Burt, J. (1992). Laser ablation of the prostate in patients with benign prostatic hyperplasia. *Brit. J. Urol.*: **69**: 603 - 608.

Holmes, S. A. V., Miller, P. D., Crocker, P. R. and Kirby, R. S. (1992). Encrustation on intraprostatic stents - A comparative study. *Brit. J. Urol.*: **69**: 379-380.

Gillatt, D., Miller, P. D., Abrams, P. and Kirby, R. S. (1992). The ASI prostatic stent: 3 years experience. *J.Urol.*: **147**: 381A.

Harrison, N. W. and De Souza, J. V. (1990). Prostatic stenting for outflow obstruction. *Brit. J. Urol.*: **65**: 192 -196.

Jones, J. P., Boullier, J. A., Rausher, J. A. and Parra, P. O. (1992). Balloon dilatation of the prostate: Lack of sustained effect with long term follow up. *J. Urol.*: **147**: 536A.

Kaplan, S. A., Soldo, K. A., Blaivas, J. G. and Olsson, C. A. (1992). Alternative therapy for symptoms of prostatism.: What do patients want ? *J. Urol.*: **147**: 539A.

Kirby, R. S., Byron, J., Eardley, I., Christmas, T. J., Liu, S., Holmes, S. A., Vale, J. A., Shanmuganathan, K. and Webb, J. A. (1992). Finesteride in the treatment of benign prostatic hyperplasia. A urodynamic evaluation. *Brit. J. Urol.*: **70**: 965 - 972.

Klein, L. A. (1992). Balloon dilatation of the prostate (BDP): Durable results. *J. Urol.*: **147**: 535A.

Lepor, H., Meretyk, S. and Knapp-Maloney, G. (1992). The safety, efficacy and compliance of Terazosin therapy for benign prostatic hyperplasia. *J. Urol.*:, **147**: 1554- 1557.

Lepor, H., Sypherd, D., Machi, E. and Derus, J., (1992). Randomised double-blind study comparing the effectiveness of balloon dilatation of the prostate and cystoscopy for the treatment of symptomatic benign prostatic hyperplasia. *J. Urol.*: **147**: 639-644.

Lewis, D. C., Burgess, N. A., Hudd, C. A. and Matthews, P. N. (1992). Open or transurethral surgery for the large prostate gland. *Brit. J. Urol.*: **69**: 598 - 602.

Ludac, R., Bloem, F. A. G. and Debruyne, F. M. J. (1992). Transurethral microwave therapy (TUMT) in benign prostatic hyperplasia. *J. Urol.*: **147**: 527A.

Matzkin, H., Soloway, M. S., Christina Rangem and Ladden, A. (1992). Efficacy of Terazosin in patients with benign prostatic hyperplasia. *Eur. Urol.*: **21**: 125 - 130.

McCullough, D. L., Winston-Salem, N. C., Roth, R. A., Burlington, M. A., Babayan, R. K., Gordon, J. O., Reece, J., Crawford, D., Fuselier, A., Krane, R. J., Assimos, D. A., Harrison, L. H., Elliot, J. P., Liam, W. H. and Daniels, G. F. (1992). TULIP - Transurethral ultrasound-guided laser induced prostatectomy National Human Cooperative study results. *J. Urol.*: **147**: 375A.

McLoughlin, J., Jager, R., Abel, P. D., El Din, A., Adam, A. and Williams G. (1990). The use of Prostatic Stents in patients with urinary retention who are unfit for surgery. *Brit. J. Urol.*: **66**: 66 -70.

McLoughlin, J., Keane, P. F., Jager, R. and Gill, K. and Williams, G. (1991). 35mm balloon dilatation of the prostatic urethra. *Brit. J. Urol.*: **67**: 177 - 181.

McLoughlin, J., Jager, R. and Williams G. (1992). Stone formation on an epithelialising prostatic stent. *Brit. J. Urol.*: **69**: 340.

Nissenkorn, I. and Richter, S. (1990). A new self - retaining Intraurethral device. An alternative to an indwelling catheter in patients with urinary retention in patients with urinary retention with infravesical obstruction. *Brit. J. Urol.*: **65**: 197 - 206.

Nissenkorn, I. and Slutzker, D. (1991). The Intraurethral Catheter: Long term follow-up in patients with urinary retention due to infravesical obstruction. *Brit. J. Urol.*: **68**: 277 - 279.

Perrin, P., Berger, C. N., Bringeon, G., Dujardin, T. and Devonec, M. (1992). Transurethral thermotherapy of benign prostatic hypertrophy: Clinical results. *J. Urol.*: **147**: 372A.

Reddy, P. K., McLeod, D. G., Wilson, S. K., Porter, A., Boileau, M. A. and Narayan, P. (1992). Experience with a 120FR balloon dilator for transurethral balloon dilatation of the prostate (BDP) for benign prostatic hypertrophy (BPH). *J. Urol.*: **147**: 534A

Rosenkilde, P., Pedersen, J. F. and Meyhoff, H. - H. (1991). Late complications of Prostakath treatment of benign prostatic hypertrophy. *Brit. J. Urol.*: **68**: 387 - 389.

Roy, J. B., Ibrahim, T. A. A. and Parry, W. L. (1992). Long term results of balloon dilatation of the prostate. *J. Urol.*: **147**, 619A.

Soldo, K. A., Shabsigh, R., Olsson, C. A. and Kaplan, S. A. (1992). Applicator-urethral distance and pre-treatment urodynamics as prognostic indicators for patients undergoing transrectal thermal therapy. *J.Urol.*: **147**: 530A.

Stoner, E. and the Finesteride study group. (1992). Clinical effects of 5 alpha reductase inhibitor Finasteride on benign prostatic hyperplasia. *J. Urol.*: **147**: 1298 -1302.

Tazaki, H. (1992). MRI valuable tool for assessing TUMT efficacy. In *Proceedings of the 22nd Congress of International Society of Urology,* Seville. p. 3.

Teillac, P., Delauche, - Cavallier, M. C., Attali, P. and the DUALF group. (1992) Urinary flow rates in patients with benign prostatic hypertrophy following treatment with Afluzosin. *Brit. J. Urol.*: **70**: 58 - 64.

Tempany, C. M. C., Zerhouni, E. A., Zinreich, S. J. and Walsh, P. C. (1992). The influence of Finesteride on the volume of the peripheral and periurethral zones of the prostate in men with benign prostatic hyperplasia. *J. Urol.*: **147**: 85A.

Van Poppel, H., Baert, L., Aswarie, H. and Ayer, R. (1991). Experience with intraprostatic coil. *Brit. J. Urol.*: **68**: 604 - 607.

Williams, G., Jager, R., McLoughlin, J., El Din, A., Machan, L., Gill, K., Asopa, R. and Adam, A. (1989). Use of prostatic stents for treating obstruction of urinary outflow in patients unfit for surgery. *Brit. Med. J.*: **298**: 1429.

Wilson, J. D. and George, F. W. (1989). An inhibitor of 5 a reductase, MK-906, suppresses prostatic dihydrotestosterone in man with prostatic hyperplasia. *J. Urol.*: **141**: 280.

Zerbib, M., Steg, A., Conquy, S., Martinache, P. R., Flam, T. A. amd Debre, B. (1992). Localised hyperthermia versus sham procedure in obstructive benign hyperplasia of the prostate: Prospective randomised study. *J. Urol.*: **147**: 1048 - 1052.

Zerbib, M., Conquy, S., Steg, A., Flam, T. and Debre, B. (1992). A three year experience with transrectal hyperthermia in the treatment of obstructive benign hyperplasia of the prostate. *J. Urol.*: **147**: 374A.

